# Epidemiology of loneliness in a cohort of UK mental health community crisis service users

**DOI:** 10.1007/s00127-019-01734-6

**Published:** 2019-06-20

**Authors:** Jingyi Wang, Brynmor Lloyd-Evans, Louise Marston, Ruimin Ma, Farhana Mann, Francesca Solmi, Sonia Johnson

**Affiliations:** 1 Division of Psychiatry, University College London, Maple House, 149 Tottenham Court Road, London, W1T 7NF UK; 2 Research Department of Primary Care and Population Health, University College London, UCL Medical School, Rowland Hill Street, London, NW3 2PF UK; 3 Camden and Islington NHS Foundation Trust, St Pancras Hospital, 4 St Pancras Way, London, NW1 0PE UK

**Keywords:** Loneliness, Prevalence, Correlates, Mental health

## Abstract

**Purpose:**

Loneliness is an important issue for mental health service users. However, it has not been a particularly prominent focus of recent mental health research. This paper aimed to explore the severity of loneliness among people leaving mental health community crisis services, and to identify factors associated with loneliness.

**Methods:**

A total of 399 participants experiencing mental health crises recruited for a research trial from community crisis services were included in this cross-sectional study. They completed the eight-item measure of the University of California at Los Angeles Loneliness Scale and a set of instruments assessing socio-demographic, psychosocial, and psychiatric variables.

**Results:**

Severity of loneliness was high among people leaving community crisis services. Longer years since first contact with mental health services (2–10 years, coefficient = 1.83, 95% CI 0.49–3.16; more than 10 years, coefficient = 1.91, 95% CI 0.46–3.36) and more severe affective symptoms (coefficient = 0.32, 95% CI 0.23–0.40) were associated with greater loneliness, whereas bigger social network size (coefficient = − 0.56, 95% CI − 0.76 to − 0.36) and greater social capital (coefficient = − 0.16, 95% CI − 0.31 to − 0.003) were associated with less severe loneliness.

**Conclusions:**

This paper supports a view that people experiencing mental health crises often report relatively severe loneliness, and that loneliness tends to become more severe during the course of illness. A greater awareness of loneliness among mental health professionals may be beneficial. Loneliness is a potential focus of the development of interventions to improve the lives and outcomes of people with significant mental health problems.

## Introduction

Loneliness can be defined as a negative emotional state that occurs when there is a subjective discrepancy between the desired and actual social relationships [1–3]. Chronic loneliness is experienced by approximately 10–15% of the general population across all ages [4]. Feelings of loneliness have been found to be more prevalent among people with mental health problems than in the general population [5, 6]. For people with depression, cross-sectional studies have found up to 40% of respondents feeling lonely all or most of the time [7], with an 11-fold increase in the odds of loneliness compared to adults with no mental disorder [8]. In a comparison between people with psychosis and a general population sample with similar demographic characteristics, the prevalence of loneliness among people with psychosis was 80% compared with 35% in the general population [9].

Evidence suggests that, in the general population, loneliness is associated with risk factors such as being a victim of domestic violence, lack of employment, not being married/partnered, being a young or an older adult (compared with middle-aged individuals), and impaired self-reported health [6, 10]. The limited available research evidence suggests that there may be some specific correlates of loneliness among people with mental health problems. Severe mental illness is known to hamper the development of social skills and negatively influence people’s ability to establish and sustain relationships [11, 12]. Depression and loneliness appear to have a circular relationship, and people with depressive symptoms sometimes have an urge to avoid social contact and isolate themselves from others [13, 14]. Stigma and social exclusion are also believed to be important causes of loneliness among people with significant mental health problems. They have an obvious effect on breaking an individual’s social ties through rejection, avoidance, and distancing from other people as well as discrimination in various areas of life [15, 16]. While some individual findings have been reported, the factors associated with loneliness have rarely been systematically studied and are not well established among people with mental health problems. Identifying these factors could help to design loneliness interventions for mental health service users in a personalised way, targeting people at particular risk of being lonely, and they may also provide pointers to potential mechanisms.

Our goal in this paper is to advance understanding of variables associated with loneliness among people experiencing mental health crises through an investigation of patterns of loneliness among people leaving the care of Crisis Resolution Teams (CRTs). CRTs, also known as home treatment teams, are a form of community crisis care intended to reduce the use of inpatient services. They offer rapid assessment to people with mental health crises and refer them to the most suitable services [17]. CRTs also provide intensive home treatment for individuals who are suitable for community-based treatment as an alternative to inpatient care [17]. CRT users are a clinically very mixed group of secondary mental health service users including a wide range of diagnoses and a mixture of long-term and short-term illness. Therefore, they represent an ideal group for researchers to begin to understand the extent of loneliness and the factors associated with it in people with relatively severe mental health problems. Our primary research aims were to: (1) assess the severity of loneliness among people leaving CRTs; and (2) to identify factors independently associated with loneliness among CRT users.

## Methods

### Study sample

The sample was taken from patients receiving care from CRTs in six NHS Trusts, which covered inner city, suburban, and more rural regions. The sample was recruited to participate in a trial of peer-delivered self-management for the CORE study (CRT Optimisation and RElapse prevention) which has already been reported [18]: this paper describes secondary analyses from the participant baseline data. The CORE study protocol was approved by the London Camden and Islington Research Ethics Committee and approvals from Research and Development departments and participating services in involved NHS Trusts were obtained. The participants had received support from a CRT team in one of the six Trusts for no less than a week and had capacity to provide written informed consent. Exclusion criteria were: (1) presenting such a high level of risk to other people that clinical staff considered that assessment on mental health service premise might not be safe for researchers, (2) not living in the catchment area, (3) not understanding English sufficiently to complete the study assessments, and (4) having been discharged from the CRT more than 1 month. After screening 3054 service users, 1697 people were eligible to participate. Among the excluded 1357 service users, 179 did not have capacity, 336 were assessed as posing too high a risk to participate, 175 were outside the recruitment area, 111 had language barriers, 121 received support from CRTs for less than 7 days, 146 were discharged from hospital rather than CRTs, and 289 had other reasons. Of the eligible participants, 401 (23.6%) were recruited and completed the assessment. The main reasons for not taking part in the study were declining to participate, not being contactable, and running out of time (interviews needed to be completed within a month of crisis team discharge). However, two participants then withdrew consent for their data to be used and so were excluded, which resulted in the study sample of 399 participants available for analyses. The sample included only those in the main trial and not the pilot sample for the CORE study.

### Measures

Here, we describe the measures used for the analyses reported in this study.

#### Loneliness

An eight-item short-form measure of the University of California at Los Angeles (UCLA) Loneliness Scale (ULS-8) [19] provided a measure of perceived loneliness. The scale was derived from the 20-item UCLA Loneliness Scale [20] by Hays and DiMatteo, and has a high level of validity and reliability [19, 21, 22]. Both the frequency and intensity of feelings of loneliness in important aspects of life are covered by the unidimensional scale (e.g., “How often do you feel that you lack companionship?”). To reduce response bias, the word ‘lonely’ never appears in the instrument [23]. Items are rated on a four-point scale: (1) Never, (2) Rarely, (3) Sometimes, and (4) Always, with higher total scores indicating greater loneliness.

#### Socio-demographic variables

Information regarding the individual’s social and demographic characteristics was recorded, including: age, gender, ethnic background, whether born in the UK, accommodation and living situation, contact with children, educational attainment, and employment.

#### Psychosocial variables

Social network size was assessed using two items from the Lubben Social Network Scale (LSNS-6) [24]. LSNS-6 is a six-item measure of social contact with family and friends, which showed high internal consistency, stable factor structures, and high correlations with criterion variables [24]. In this paper, we used the two scale items which assessed amount of social contact with family and friends (total score 0–10; higher total score indicating larger social network size), ignoring the other four items which were related to the perceived quality of social relationships and overlapped conceptually with loneliness. Social capital was assessed with the Health and Lifestyles Survey Social Capital Questionnaire [25], a six-item measure of neighbourhood social capital regarding enjoyment of living, personal safety, neighbours looking after each other, facilities for children, local transport, and leisure facilities. This gives a minimum social capital score of -6 and a maximum of 6, with higher total scores indicating greater neighbourhood social capital.

#### Psychiatric variables

The Brief Psychiatric Rating Scale (BPRS 4.0) [26], a 24-item scale of psychiatric symptom severity, was used to rate participants’ present condition. Possible scores vary from 24 to 168 with lower scores indicating less severe psychopathology. Good-to-excellent inter-rater reliability of the BPRS has been confirmed in the existing studies [26–29]. Following guidance based on factor analysis of the BPRS [30], three subscales were derived for analyses in this paper due to their potential relevance to loneliness: affect subscale (anxiety, depression, suicidality, and guilt), positive symptoms subscale (grandiosity, suspiciousness, hallucinations, and unusual thought content), and negative symptoms subscale (blunted affect, emotional withdrawal, and motor retardation). In addition, number of psychiatric inpatient hospitalisations in a lifetime, number of years, since first contact with mental health services, and participants’ baseline clinical diagnoses were collected.

### Procedures

Potentially eligible participants were initially identified by clinicians in CRTs. They were then contacted by clinical staff in CRTs or other community mental health services to introduce the study and establish willingness to be called by study researchers for further discussion. Those who were willing to be contacted by researchers were informed of the content and procedures of the study and were invited to participate. All participants provided their informed consent. After written consent was received, study baseline measures were completed with all consenting participants as a structured interview. Diagnoses of mental health problems were collected from patient clinical records by researchers at the end of recruitment.

### Statistical analyses

All data were checked for missing values. The loneliness scale (ULS-8) was completed by all the respondents with 8 (2.0%) having 1–2 items missing. The number of participants who had missing items on the other measures ranged from 1 (0.3%) to 7 (1.8%). As the percentage of missing data was low, case mean substitution was used to replace the missing items with the subject’s mean score based on the items that were present for that subject [31]. This method was only used for ULS-8, BPRS subscales, and social capital, as this technique is appropriate to measures where all items are indicators of a specific concept or construct [31] and the items are parallel and approximately interchangeable [32]. As case mean substitution was reported not to result in highly biased estimates only when less than 25% of item scores and less than 10% of cases are missing [33], 8 cases with more than 25% missing data on BPRS subscales or social capital measures were removed from the corresponding analyses.

Descriptive statistics were obtained for all variables. For variables not normally distributed, medians and interquartile ranges were reported; otherwise, means and standard deviations were reported. With respect to categorical variables, descriptive statistics were reported as frequencies and percentages within each category. A series of univariate linear regression analyses were carried out with baseline loneliness score as dependent variable and the independent variables separately. The residuals of the models were checked; the models were good fit, so variable transformations were not conducted.

Three multivariable linear regression models were carried out to examine the association between loneliness scores and the three sets of explanatory variables. Blocks of independent variables associated at the *p *< 0.25 level with the outcome variable in univariate linear regression were entered into the multivariable regression models in the following order: (1) socio-demographic variables (age, whether born in the UK, accommodation, employment, and living situation); (2) socio-demographic and psychosocial variables (social network size and social capital); and (3) socio-demographic, psychosocial, and psychiatric variables (number of psychiatric inpatient hospitalisations, number of years since first contact with mental health services, affective symptoms, positive symptoms, negative symptoms, and clinical diagnoses). Psychosis was used as a reference in clinical diagnoses, because the majority of CRT users are people with psychosis, and its association with loneliness may be different from other affective disorders.

All analyses were performed using Stata version 12.1. *P* values of less than 0.05 were considered significant.

## Results

### Sample characteristics

The sample at baseline consisted of 399 participants (40.2% male). The median age was 40.0 (IQR 29.9–50.0), with the youngest respondent being 18 years of age and the oldest 75. 53.8% of participants were White British. A large proportion of the sample, 89.7%, had independent accommodation, 46.5% were currently living with a partner or with family, and 16.8% were living with dependent children. In terms of education and employment, 27.4% had a degree, whilst 27.3% were in regular employment. More than one in four participants had a diagnosis of schizophrenia or psychosis (27.0%), 16.3% were diagnosed with bipolar affective disorder/manic episode, 35.0% suffered from depressive/anxiety disorders, 13.3% from personality disorders, and 8.4% from other disorders. The characteristics of respondents are described in Table 1.

**Table 1:** Socio-demographic, psychosocial, and psychiatric characteristics of crisis resolution team users

Characteristic	*N* (%) or mean (SD) or median (IQR)
Age (years)	40.0 (29.9–50.0)
Gender	
Male	160 (40.2%)
Female	238 (59.8%)
Ethnic background	
White British	214 (53.8%)
White other	40 (10.1%)
Black/Black British	80 (20.1%)
Asian/Asian British	37 (9.3%)
Mixed	27 (6.8%)
Born in the UK	
No	89 (22.7%)
Yes	304 (77.4%)
Housing	
Independent accommodation	357 (89.7%)
Other	41 (10.3%)
Contact with children under 16	
Living with dependent children	67 (16.8%)
Other	332 (83.2%)
Education attainment	
No qualifications	76 (19.1%)
Other qualifications	213 (53.5%)
Degree	109 (27.4%)
Employment	
No	257 (64.4%)
In voluntary, protected, or sheltered work	33 (8.3%)
In regular employment	109 (27.3%)
Living with a partner or with family	
No	213 (53.5%)
Yes	185 (46.5%)
Loneliness (8–32)	22 (19–25)
Social network size (0–10)	4.9 (2.3)
Social capital (− 6 to 6)	3 (0–5)
Number of psychiatric inpatient hospitalisations	
Never	148 (37.1%)
Once	86 (21.6%)
2–5 times	102 (25.6%)
More than 5 times	63 (15.8%)
Number of years since first contact with mental health services	
Less than 3 months	67 (16.8%)
3 months–1 year	39 (9.8%)
1–2 years	28 (7.0%)
2–10 years	126 (31.7%)
More than 10 years	138 (34.7%)
Affective symptoms (4–28)	12 (8–17)
Positive symptoms (4–28)	5 (4–8)
Negative symptoms (3–21)	4 (3–6)
Clinical diagnosis	
Psychosis (schizophrenia and other non-affective psychosis)	106 (27.0%)
Bipolar affective disorder/manic episode	64 (16.3%)
Depressive/anxiety disorders	137 (35.0%)
Personality disorders	52 (13.3%)
Other disorders	33 (8.4%)

For instruments (loneliness, social network size, social capital, affective symptoms, positive symptoms, and negative symptoms), range of scores is indicated between brackets

*N* number of participants, *SD* standard deviation, *IQR* interquartile range

### Severity of loneliness at baseline among people leaving CRTs

The descriptive statistics for ULS-8 items are summarised in Table 2. Over 70% of participants sometimes or always felt that they lacked companionship, left out, isolated from others, unhappy being so withdrawn, and that people were around them but not with them. Data about the frequency and intensity of loneliness experiences were summed to provide a total loneliness score from 8 to 32. The mean total score was 21.9 (SD = 5.0); the total score exhibited slight negative skew. Thus, the median score, 22 (IQR 19–25), is reported in Table 1. Although there is no standard threshold for severe loneliness for ULS-8, a score of 24 is a potential cut-off as it equates to answering “sometimes” to every question. In our sample, 30.6% of participants had a total score over 24 and could be considered as having severe loneliness.

**Table 2:** Descriptive statistics for ULS-8 items

Item	Never *N* (%)	Rarely *N* (%)	Sometimes *N* (%)	Always *N* (%)
1. How often do you feel that you lack companionship?	40 (10.0)	57 (14.3)	205 (51.4)	97 (24.3)
2. How often do you feel that there is no one you can turn to?	60 (15.0)	66 (16.5)	195 (48.9)	78 (19.6)
3. How often do you feel that you are an outgoing person?	59 (14.8)	108 (27.1)	164 (41.1)	68 (17.0)
4. How often do you feel left out?	41 (10.3)	73 (18.3)	194 (48.6)	91 (22.8)
5. How often do you feel isolated from others?	32 (8.0)	61 (15.3)	199 (49.9)	107 (26.8)
6. How often can you find companionship when you want it?	37 (9.3)	71 (17.8)	179 (44.9)	112 (28.1)
7. How often do you feel unhappy being so withdrawn?	34 (8.5)	57 (14.3)	176 (44.1)	132 (33.1)
8. How often do you feel people are around you but not with you?	35 (8.8)	55 (13.8)	195 (48.9)	114 (28.6)

*ULS-8* UCLA Loneliness Scale-8, *N* number of participants

### Factors associated with loneliness in people experiencing mental health crises

The results of univariate linear regression analyses investigating factors associated with baseline loneliness are shown in Table 3. Feelings of loneliness were more intense in people for whom it had been 2–10 years since first contact with mental health services compared to those in contact with services for less than 3 months. Greater severity of loneliness was also associated with more severe affective symptoms, positive symptoms or negative symptoms, and with diagnosis of depressive/anxiety disorder, personality disorder, or other disorders as opposed to psychosis. Lower levels of loneliness were associated with bigger social network size, more neighbourhood social capital, and having been hospitalised more than once (with the never hospitalised as a reference group).

**Table 3:** Results of univariate linear regression analyses for factors associated with baseline loneliness

Variables	Coefficient^a^	95% CI	*P* value^b^
*Socio-demographic variables*			
Age (years)	− 0.03	− 0.07 to 0.01	0.11
Gender (0 = male; 1 = female)	0.39	− 0.62 to 1.39	0.45
Ethnic background			
White British	Reference		
White other	− 0.13	− 1.82 to 1.57	0.88
Black/Black British	− 0.44	− 1.73 to 0.85	0.50
Asian/Asian British	0.62	− 1.13 to 2.37	0.49
Mixed	− 0.83	− 2.84 to 1.18	0.42
Born in the UK (0 = no, 1 = yes)	1.13	− 0.04 to 2.31	0.06
Housing (0 = other, 1 = independent accommodation)	− 1.08	− 2.70 to 0.53	0.19
Contact with children under 16 (0 = other; 1 = living with dependent children)	− 0.73	− 2.05 to 0.58	0.27
Education attainment			
No qualifications	Reference		
Other qualifications	− 0.11	− 1.42 to 1.20	0.87
Degree	0.23	− 1.23 to 1.70	0.76
Employment			
No	Reference		
In voluntary, protected or sheltered work	− 0.25	− 2.06 to 1.56	0.79
In regular employment	− 1.00	− 2.12 to 0.12	0.08
Living with a partner or with family (0 = no; 1 = yes)	− 0.94	− 1.93 to 0.04	0.06
*Psychosocial variables*			
Social network size (two items from LSNS-6)	− 0.79	− 1.00 to − 0.59	**< 0.001**
Social capital (HLSSC)	− 0.52	− 0.68 to − 0.36	**< 0.001**
*Psychiatric variables*			
Number of psychiatric inpatient hospitalisations			
Never	Reference		
Once	− 1.29	− 2.60 to 0.03	0.06
2–5 times	− 1.46	− 2.71 to − 0.21	**0.02**
More than 5 times	− 2.13	− 3.59 to − 0.67	**0.004**
Number of years since first contact with mental health services			
Less than 3 months	Reference		
3 months–1 year	1.37	− 0.59 to 3.34	0.17
1–2 years	1.80	− 0.39 to 4.00	0.11
2–10 years	1.58	0.10 to 3.05	**0.04**
More than 10 years	1.23	− 0.23 to 2.68	0.10
Affective symptoms (four items from BPRS)	0.45	0.38 to 0.53	**< 0.001**
Positive symptoms (four items from BPRS)	0.24	0.12 to 0.35	**< 0.001**
Negative symptoms (three items from BPRS)	0.38	0.17 to 0.60	**0.001**
Clinical diagnosis			
Psychosis (schizophrenia and other non-affective psychosis)	Reference		
Bipolar affective disorder/manic episode	− 0.85	− 2.37 to 0.67	0.27
Depressive/anxiety disorders	1.97	0.73 to 3.21	**0.002**
Personality disorders	2.60	0.98 to 4.23	**0.002**
Other disorders	1.93	0.01 to 3.84	**0.048**

*CI* confidence interval, *LSNS-6* Lubben Social Network Scale-6, *HLSSC* Health and Lifestyles Survey Social Capital Questionnaire, *BPRS* Brief Psychiatric Rating Scale

^a^Negative regression coefficient = less loneliness

^b^Significant *p* values printed in bold

Table 4 presents the results of multivariable linear regression analyses for factors associated with baseline loneliness. Model 1, including only socio-demographic variables, explained 2.0% of the variance in loneliness. The only factor significantly associated with greater loneliness was younger age. After adding psychosocial variables to socio-demographics in model 2, the amount of variance explained rose to 18.1%. In this model, greater loneliness was related to younger age, being born in the UK, smaller social network size, and lower neighbourhood social capital. Model 3, where all the three blocks of independent variables were entered into the regression equation, explained a total of 36.2% of the variation in loneliness. In this final model, more severe feelings of loneliness proved to be significantly associated with more than 2 years since first contact with mental health services (compared with fewer than 3 months), and with more severe affective symptoms. Lower levels of loneliness were associated with bigger social network size, greater neighbourhood social capital, and more than 5 psychiatric inpatient hospitalisations compared with no hospitalisations. After adjusting for clinical variables, the association of loneliness with age was no longer significant and the association with being born in the UK became borderline significant.

**Table 4:** Results of multivariable linear regression analyses for factors associated with baseline loneliness^a^

Variables	Model 1			Model 2			Model 3		
	Coefficient^b^	95% CI	*P* value^c^	Coefficient	95% CI	*P* value	Coefficient	95% CI	*p* value
*Socio-demographic variables*									
Age (years)	− 0.04	− 0.08 to − 0.001	**0.045**	− 0.05	− 0.09 to − 0.02	**0.01**	− 0.03	− 0.06 to 0.01	0.17
Born in the UK (0 = no; 1 = yes)	0.98	− 0.21 to 2.16	0.11	1.12	0.03 to 2.22	**0.045**	1.00	− 0.02 to 2.02	0.05
Housing (0 = other; 1 = independent accommodation)	− 0.56	− 2.21 to 1.09	0.51	0.12	− 1.42 to 1.66	0.88	0.45	− 1.00 to 1.90	0.54
Employment									
No	Reference								
In voluntary, protected, or sheltered work	− 0.32	− 2.16 to 1.52	0.73	0.23	− 1.45 to 1.90	0.79	0.66	− 0.85 to 2.17	0.39
In regular employment	− 1.00	− 2.15 to 0.15	0.09	− 0.04	− 1.11 to 1.03	0.94	− 0.16	− 1.19 to 0.87	0.76
Living with a partner or with family (0 = no; 1 = yes)	− 0.87	− 1.89 to 0.15	0.10	− 0.36	− 1.30 to 0.58	0.46	− 0.47	− 1.37 to 0.42	0.30
*Psychosocial variables*									
Social network size (two items from LSNS-6)				− 0.71	− 0.93 to − 0.50	**< 0.001**	− 0.56	− 0.76 to − 0.36	**< 0.001**
Social capital (HLSSC)				− 0.37	− 0.53 to − 0.20	**< 0.001**	− 0.16	− 0.31 to − 0.003	**0.046**
*Psychiatric variables*									
Number of psychiatric inpatient hospitalisations									
Never							Reference		
Once							− 0.97	− 2.09 to 0.16	0.09
2–5 times							− 1.21	− 2.49 to 0.06	0.06
More than 5 times							− 1.82	− 3.38 to − 0.26	**0.02**
Number of years since first contact with mental health services									
Less than 3 months							Reference		
3 months–1 year							1.39	− 0.30 to 3.09	0.11
1–2 years							1.91	− 0.01 to 3.82	0.05
2–10 years							1.83	0.49 to 3.16	**0.01**
More than 10 years							1.91	0.46 to 3.36	**0.01**
Affective symptoms (four items from BPRS)							0.32	0.23 to 0.40	**< 0.001**
Positive symptoms (four items from BPRS)							0.10	− 0.01 to 0.21	0.07
Negative symptoms (three items from BPRS)							0.18	− 0.02 to 0.38	0.07
Clinical diagnosis									
Psychosis							Reference		
Bipolar affective disorder/manic episode							− 0.60	− 2.00 to 0.80	0.40
Depressive/anxiety disorders							0.95	− 0.39 to 2.29	0.17
Personality disorders							− 0.01	− 1.47 to 1.46	0.99
Other disorders							0.39	− 1.41 to 2.18	0.67
$R^{2}_{\text{adj}}$	0.020			0.181			0.362		

*CI* confidence interval, *LSNS-6* Lubben Social Network Scale-6, *HLSSC* Health and Lifestyles Survey Social Capital Questionnaire, *BPRS* Brief Psychiatric Rating Scale, $R^{2}_{\text{adj}}$ = adjusted-*R*^2^

^a^Using multivariable linear regression analyses with loneliness score at baseline as dependent variable and factors with *p *< 0.25 in univariate linear regression as independent variables

^b^Negative regression coefficient = less loneliness

^c^Significant *p* values printed in bold

## Discussion

When compared with studies which used the same loneliness scale, levels of loneliness in this sample of CRT users seemed to be considerably greater among people experiencing mental health crises (*M* = 21.9, SD = 5.0) than among young adults in two community-based samples of 19–39 years old from the 1996 Niagara Young Adult Study (*M* 15.78–16.08 and SD 5.08–5.27) [34] and Italian community-dwelling older adults of 65–89 years old from the Act on Ageing project (*M* = 13.1, SD = 6.9) [35], although their age ranges did not match the ages of our sample (18–75 years old). Since the mean score for loneliness in our sample was much higher than that in the two general population samples mentioned and this finding was consistent with existing research [9, 36], it is reasonable to conclude that feelings of loneliness were more prevalent amongst mental health community crisis service users than in the general population. In mental health context, the severity of loneliness in our sample (*M* = 21.9, SD = 5.0) was comparable to adults with social anxiety disorder (*M* 23.68–25.07 and SD 2.79–4.73) [37] and adults with autism spectrum disorders (*M* = 20.9 and SD = 4.7) [38].

A second aim of this paper was to identify factors independently associated with loneliness among individuals experiencing mental health crises. In the first multivariable regression model with socio-demographic characteristics as explanatory variables, younger age was associated with increased loneliness. This result coheres with the BBC’s Loneliness Experiment which reported 16–24 years old as the group who feel loneliest among 55,000 members of the British public [39]. However, only 2% of the variance in loneliness was explained in the first model, and in the final model, loneliness did not have significant association with any of the socio-demographics after adjusting for psychosocial and psychiatric variables. This finding is consistent with the previous research in people with psychosis [9, 36, 40], although socio-demographic characteristics have been reported as linked to individual differences in loneliness in studies of general population [41, 42]. It is possible that the social impact of having a significant mental health problem is sufficiently severe that it overrides other socio-demographic factors which might, otherwise, be expected to make a difference in loneliness.

As psychosocial variables, social network size and neighbourhood social capital were found to be inversely related to the severity of loneliness, explaining 18.1% of the variance in loneliness together with socio-demographic factors. The finding coheres with the existing studies showing that objective measures of social relations and perception of community-level structures or characteristics may affect the severity of loneliness [43, 44], although the direction of causality cannot be inferred. Communities with poor social capital may predispose an individual to loneliness, or lonely people may find it more difficult to recognise community social resources. As the proportion of variance explained is relatively low, the findings also suggest that there are some major influences on loneliness that are not captured by social network size and neighbourhood social capital.

After adding psychiatric variables to the final regression model, the explained variance in loneliness increased to 36.2%. It is surprising that having had more than five psychiatric admissions was associated with less severe loneliness. This result is contradictory to the previous research which reported that a greater number of psychiatric inpatient admissions were related to more intense feelings of loneliness [36]. As we explored associations between loneliness and many variables, this may be just a chance finding. It is also possible that peer support developed during psychiatric hospitalisations and, perhaps, lasted beyond admission, or that having multiple admissions might be a marker for a group of people who were relatively intensively engaged with services and so more likely to have their needs addressed. If this finding is replicated in other studies, future research could include mixed method exploration of whether there are potential mechanisms to underpin an association between hospitalisations and social support and loneliness. It is more intuitively understandable that longer years since first contact with mental health services were related to greater loneliness. People may withdraw from social connections or lose intimate or confiding relationships cumulatively through the course of an enduring mental illness. It may also be that people’s positive social identities and hopefulness about restoring relationships erode over time, which may increase feelings of loneliness. The emotional impact of relapse may be greater every time people experience mental health relapse and this may impact on feelings of loneliness. More severe affective symptoms (a summary score including symptoms of anxiety, depression, suicidality, and guilt) were associated with greater loneliness, while severity of positive symptoms or that of negative symptoms of psychosis had no association with loneliness. A recent systematic review found contradictory results about the relationship between loneliness and psychotic symptomatology [45], but findings from a meta-analysis reported a moderate association between loneliness and psychosis [46]. The relationship between depressive symptoms and loneliness was stronger and more consistent in the previous studies. Adults with depressive episode were around 11 times more likely to feel lonely compared to those with no mental disorder [8]. Greater loneliness was also reported to be associated with more severe depressive symptoms and poorer remission from depression in a systematic review [47]. Masi and colleagues [48] described a regulatory loop model of loneliness, in which people who are lonely tend to have negative and biased social cognitions [49]. These cognitions are likely to get people involved in behavioural confirmation processes which generate more negative social interactions and elicit further confirmation of their poor social value, and, finally, result in greater loneliness [48, 49]. People with depression also tend to have negative bias in thinking processes which leads to negative patterns of behaviour [50]. The two factors together with biological processes, stressors, and interpersonal factors interact with one another and form a “negative downward loop” which pushes people further into depression [51]. Therefore, a plausible hypothesis is that loneliness and depression may be involved in a double feedback loop reinforcing each other [52]. In the multivariable model, loneliness was not associated with participants’ diagnoses. A possible explanation is that the impact of these diagnoses on loneliness was largely due to the differences in severity of symptoms. We used the BPRS to rate participants’ current symptoms which appeared to have a greater effect on feelings of loneliness than their long-term diagnoses.

### Limitations of the study

The results of this study should be interpreted with a number of limitations in mind. First, the cross-sectional design of this paper makes it impossible to examine causal relationships between loneliness and its correlates. Second, the participants were assessed immediately post-crisis which is a very particular time. They may experience more severe loneliness during the long-term recovery journey than during crisis. Third, the recruitment rate of 23.6% of identified eligible potential participants was low and might have resulted in some degree of selection bias. The low recruitment rate in our sample may be related to the mental health crises that the service users had just experienced before recruitment, which leads to the concern that people with severe symptoms may disproportionately fall into the nonresponse group, and, thus, results should be interpreted with some caution. Finally, our sample may underrepresent people who were unwilling to participate in a trial generally, or to whom the specific intervention which we were offering did not appeal. This might introduce some biases that might be absent in a straightforward epidemiological study.

### Clinical and policy implications

The present study has some important implications for practice and policy. The high prevalence of loneliness is a serious problem for which we need to seek solutions considering its adverse physical and mental health consequences as well as its impact on resource use [47, 53]. Although loneliness is common in mental health community crisis service users, it may be overlooked in clinical environment due to lack of awareness among practitioners, or due to the practitioners feeling, they may not be able to address any needs identified, or avoiding the subject for fear it will be perceived as intrusive or upsetting by the service users. They may also not consider that it is their responsibility to help with this [54]. It may be helpful for mental health professionals to screen for the severity of loneliness to identify people at risk, given that loneliness may influence mental health in both immediate and long term [8, 36, 55, 56]. The study also showed that individuals with severe affective symptoms or with a long history of mental illnesses were especially prone to loneliness and thus need further attention from clinicians. A validated short screening tool can potentially provide practitioners a time and cost-effective approach to identify people in need, e.g., the De Jong Gierveld 6-Item Loneliness Scale [57] and the UCLA 3-Item Loneliness Scale [58] which are short and possess robust psychometric properties. Evaluation is helpful to show that practitioners are sincerely helping people who turn to the services and even a simple question asking people whether they are lonely may make an impact.

It is important to create a culture in mental health services where loneliness is viewed as a valid area to discuss and try to tackle, and for practitioners to work with service users to maximise available support from families and local community resources to address loneliness. More importantly, the high prevalence of loneliness among service users is not only an individual but also a community and societal level problem, and macrosocial factors have been proposed as significant determinants of levels and content of social relationships [59]. Therefore, it is imperative to advance public awareness of the importance of reducing loneliness, and ultimately to urge policy level changes to support disadvantaged populations. The Campaign to End Loneliness took the first steps towards diminishing loneliness and inspiring national, regional, and local organisations, and people to deal with its health threat in older age [60]. The Campaign utilises a partnership approach, and joint projects are delivered by various organisations to raise awareness of loneliness, identify and develop effective loneliness interventions, and establish a reliable lifelong network of support [60]. However, the Campaign did not specifically focus on mental health service users and has not been robustly evaluated. Therefore, interventions targeting mental health service users need to be developed and tested, considering the great social impact of having mental health problems. A similar collaborative approach could be conducted to inspire organisations and people to tackle loneliness issues as a mental health priority at the level of communities and societies, which might be the most effective approach. Nonetheless, we would need to be confident that mental health service users, for whom stigma and social exclusion are problems, will also benefit from similar approaches designed for the general population.

### Research implications

In terms of research implications, scientists should be trying to understand the phenomenon of loneliness and potential strategies to address it more from service users’ perspective. For example, the most widely used loneliness measures were initially designed for the general population [61, 62]. We cannot be sure whether these measures are perceived by service users as a meaningful reflection of their experiences. Perhaps, new measures, respectively, targeting children/young people and adults could be produced and interventions could be designed in collaboration with service users. In addition, more longitudinal research is needed to untangle the direction of effect in the relationship between loneliness and poor outcomes as well as the mechanisms through which loneliness affects mental health. We also need more longitudinal research to understand the course of loneliness for people with severe mental illness. Loneliness is not such a serious problem if it is short term: this study suggests that loneliness may typically get worse during the course of someone’s contact with mental health services, but understanding the extent of chronic loneliness and the characteristics of those affected by mental health crises is necessary to inform appropriate interventions.

To conclude, this study supports a view that people experiencing mental health crises have more severe loneliness than the general population. The quantitative investigation also highlighted the strong association between greater loneliness and small social network size, limited social capital, more severe affective symptoms, and long-term mental illness history. Therefore, interventions targeting loneliness and mental health care professionals should pay more attention to people at particular risk of being lonely.
